# Ptch2 is a Potential Regulator of Mesenchymal Stem Cells

**DOI:** 10.3389/fphys.2022.877565

**Published:** 2022-04-28

**Authors:** Emma Juuri, Pauli Tikka, Andrii Domanskyi, Ian Corfe, Wataru Morita, Peter J. Mckinnon, Nela Jandova, Anamaria Balic

**Affiliations:** ^1^ Cell and Tissue Dynamics Research Program, Institute of Biotechnology, HiLIFE, University of Helsinki, Helsinki, Finland; ^2^ Orthodontics, Oral and Maxillofacial Diseases, University of Helsinki, Helsinki, Finland; ^3^ Oral and Maxillofacial Diseases, Helsinki University Hospital, Helsinki, Finland; ^4^ Circuar Economy Solutions Unit, Geological Survey of Finland, Espoo, Finland; ^5^ Department of Anthropology, National Museum of Nature and Science, Taito, Japan; ^6^ Department of Genetics, St. Jude Children’s Research Hospital, Memphis, TN, United States; ^7^ Department of Experimental Biology, Faculty of Science, Masaryk University, Brno, Czechia; ^8^ Institute of Animal Physiology and Genetics, CAS, Brno, Czechia; ^9^ Institute of Oral Biology, Centre for Dental Medicine, University of Zürich, Zürich, Switzerland

**Keywords:** Ptch2, Hedgehog pathway, Gli1, Ptch1, Gli2, stem cells

## Abstract

Ptch receptors 1 and 2 mediate Hedgehog signaling pivotal for organ development and homeostasis. In contrast to embryonic lethal *Ptch1* ^−/−^ phenotype, *Ptch2* ^*−/−*^ mice display no effect on gross phenotype. In this brief report, we provide evidence of changes in the putative incisor mesenchymal stem cell (MSC) niches that contribute to accelerated incisor growth, as well as intriguing changes in the bones and skin which suggest a role for Ptch2 in the regulation of MSCs and their regenerative potential. We employed histological, immunostaining, and computed tomography (µCT) analyses to analyze morphological differences between *Ptch2* ^*−/−*^ and wild-type incisors, long bones, and skins. *In vitro* CFU and differentiation assays were used to demonstrate the MSC content and differentiation potential of *Ptch2* ^*−/−*^ bone marrow stromal cells. Wound healing assay was performed *in vivo* and *in vitro* on 8-week-old mice to assess the effect of Ptch2 on the wound closure. Loss of *Ptch2* causes increases in the number of putative MSCs in the continuously growing incisor, associated with increased vascularization observed in the tooth mesenchyme and the neurovascular bundle. Increased length and volume of *Ptch2* ^*−/−*^ bones is linked with the increased number and augmented *in vitro* differentiation potential of MSCs in the bone marrow. Dynamic changes in the *Ptch2* ^*−/−*^ skin thickness relate to changes in the mesenchymal compartment and impact the wound closure potential. The effects of *Ptch2* abrogation on the postnatal MSCs suggest a crucial role for Ptch2 in Hedgehog signaling regulation of the organ regenerative potential.

## Introduction

The Hedgehog (Hh) pathway plays a crucial role in the regulation of cell-fate specification, differentiation, tissue homeostasis, and in development of pathological conditions such as cancer. Hh signaling is initiated upon binding of the Hh ligand to receptors Ptch1 or Ptch2 (Kong et al., 2019). This releases transmembrane protein Smoothened (Smo) and triggers nuclear translocation of Gli1-3 proteins (Hui and Angers, 2011).

Mouse Ptch2 shares 56% homology with Ptch1 (Motoyama et al., 1998) and directly antagonizes Hh ligand function, thus limiting pathway activation and Smo activity (Holtz et al., 2013; Alfaro et al., 2014; Roberts et al., 2016). Previous reports suggest that Ptch2 and Ptch1 are functionally redundant (Adolphe et al., 2014; Zhulyn et al., 2015). However, contrasting phenotypes of embryonic lethal *Ptch1* ^*−/−*^ animals (Goodrich et al., 1997) and grossly normal *Ptch2* ^*−/−*^ mice (Lee et al., 2006) have provided the basis for the current view that Ptch2 plays a minor role in Hh signaling. This is further supported by subtle gross phenotypic changes in mice lacking *Ptch2* and hypomorphic *Ptch2* mutants (Lee et al., 2006; Nieuwenhuis et al., 2006; Holtz et al., 2013; Klein et al., 2016).

We recently demonstrated that multimodal Hh signaling regulation of the cellular hierarchy in the mouse incisor tooth epithelial stem cell (SC) niche is enabled by the functional differences between the Ptch1 and Ptch2 receptors (Binder et al., 2019), which were also observed in the germ cell niche (Kim et al., 2020). In addition, loss of *Ptch2* enhanced regeneration of tooth mineralized matrices, which suggests that Ptch2 is a critical regulator of SC differentiation (Binder et al., 2019). Furthermore, these studies implied an independent role for the Ptch2 receptor in the inhibition of Hh signaling.

In this study, we analyzed several organs obtained from mice lacking *Ptch2* (*Ptch2* ^−/−^), including teeth, long bones, and skin. We observed intriguing changes in tissues in which Ptch2 is co-expressed with Ptch1, that affect the mesenchymal stem cells (MSCs) and their regenerative and differentiation potential. These subtle, yet intriguing changes indicate an independent role for Ptch2 and suggest that absence of Ptch2 correlates with increased SC function. In addition, we provide evidence that suggests that Ptch2 transduces Hh signaling mainly through regulation of Gli1 protein. Taken together, our data provide compelling evidence that Ptch2 regulates SC function and postnatal regenerative capacity.

## Materials and Methods

### Mice

In this study we used *Ptch2* ^*−/−*^ mutant mice (Lee et al., 2006). All experimental procedures involving mice were approved by the Ethical Committees on the Use and Care of Animals and the Animal Facility at the University of Helsinki.

### Skin Wound Healing

All animals (3 per genotype) were anesthetized prior the procedure. Analgesics were administered prior to wounding and for 3 consecutive days after wounding. Two to four full-thickness wounds were made on the dorso-rostral skin using a 4-mm biopsy punch as previously described (Aragona and Blanpain, 2017). Mice were sacrificed 7 days post-wounding and tissue was processed for histological and immunostaining analyses.

### Tissue Isolation and Preparation

Mouse mandibles and neurovascular bundles (NVBs) were isolated from 3-4-week-old mice following an established protocol (Balic, 2019). Dorsal skins were collected from postnatal animals at days 1, 14, and 50. All samples were fixed with 10% formalin overnight at 4°C. Mandibles and skins were dehydrated, embedded in paraffin wax, and sectioned into 7–12 μm sections. NVBs were processed for whole-mount immunofluorescent staining. Long bones were collected from 4-5-week-old mice, cleaned from the surrounding tissue, and kept in 100% ethanol until µCT scanning.

### Cells and Cell Cultures

*MEFs* obtained from E14.5 wild-type and *Ptch2* ^*−/−*^ (Lee et al., 2006), and E10.5, *Ptch1* ^*−/−*^ embryos (gift from Dr. Ben Allen), were plated on chamber slides with removable wells at a density of 10,000 cells/0.1 cm^2^. Culture media were used as previously described (Binder et al., 2019). The following day, cultures were washed and processed for immunofluorescent staining.

Bone marrow stromal cells were isolated from the metaphyses of cleaned tibiae and cultured as previously described (Balic et al., 2010). CFU assay was performed as previously described (Yang et al., 2013). ImageJ was used to quantify the number of colonies formed after 7 days of culture. Data are represented as relative values normalized against wild-type control in each experimental replicate.

Mineralization and adipogenesis were induced in confluent cultures at day 7 or 10, respectively, as previously described (Balic et al., 2010). Alizarin Red staining (AR-S) was used to assay the extent of mineralization in terminated cultures. Adipocytes were visualized by Nile Red staining of live cultures. Briefly, cultures were incubated with Nile Red (1:1000 dilution of stock solution) for 1 h at 37°C, washed, and imaged under a fluorescent microscope. ImageJ was used to quantify the surface of AR-S and Nile Red staining. Data are represented as relative values normalized against wild-type control in each experimental replicate.

### Immunostainings

#### Cell Cultures

One day after plating, cultures were washed with PBS and fixed with 10% formalin for 5 min at room temperature followed by a 5-min wash with 100% methanol and subsequent washes with PBS. Fixed samples were then permeabilized with 0.1% Triton in PBS for 5 min, blocked for 1 h in 10% donkey serum, 1% BSA, 0.1% Triton in PBS, and incubated with primary antibody at room temperature for 2 h. Secondary antibodies were incubated for 45 min to 1 h at room temperature. After extensive washes with PBS, the samples were mounted using Vectashield with DAPI (Vector Laboratories).

#### Tissue Sections

Prior to permeabilization with 0.1–0.3% Triton, deparaffinized and rehydrated paraffin sections were incubated with TE buffer, pH 9 at 95°C for 10 min for antigen retrieval. After blocking for 1 h in 10% donkey serum (Sigma-Aldrich), 1% BSA, 0.1–0.3% Triton in PBS, the sections were incubated overnight at 4°C with the first primary antibody (Table 1). The following day, samples were washed with PBS and incubated with the second primary antibody for 2 h at room temperature. Samples were then incubated with secondary antibodies at room temperature for 45 min to 1 h, washed with PBS, and mounted using Vectashield with DAPI.

**TABLE 1:** List of primary antibodies and dilutions used in the study.

Antibody	Dilution	Catalog number/Manufacturer
CD146	1:1000	134706/BioLegend, San Diego, California, United States
CD31	1:500	102507/BioLegend, San Diego, California, United States
Gli1	1:100	NBP1-78259/Novus, Littleton, Colorado, United States
Gli2	1:250	NB600-874SS/Novus, Littleton, Colorado, United States
Gli3	1:100	AF3690/R&D, Minneapolis, Minnesota, United States
MBP	1:2000	40390/Abcam, Cambridge, United Kingdom
Keratin-14	1:400	RB9020P/ThermoFisher, Waltham, Massachusetts, United States
Ptch1	1:200	109096/Abcam, Cambridge, United Kingdom
Ptch2	1:200	2470/Cell Signalling Technology, Danvers, Massachusetts, United States
Lhx2	1:500	sc-19344/Santa Cruz, Dallas, Texas, United States

#### Whole Mount

Immunostaining of neurovascular bundles was performed following the established protocol for organ culture whole mount staining (Binder et al., 2019).

### Skin Morphometric Analyses

H&E stained skin sections were visualized using high-throughput brightfield scanning microscope equipped with 4 MP CMOS camera with 130 fps speed. Slides were scanned with extended focus (Z-stack) with 0.24 μm/pixel resolution with the combined 20X/0.8 NA objective (equivalent to ×40 magnification). Fixed-size images (2.5 × 1 mm) of random sections were collected for each sample (minimum 5 sections) using Case viewer software (3DHISTECH). Skin area in each image was calculated using ImageJ.

### Quantification of Nuclear Immunofluorescent Intensity

Cultures of wild-type, *Ptch1* ^*−/−*^, and *Ptch2* ^*−/−*^ MEFs were immunostained against Gli1, Gli2, or Gli3 protein. DAPI staining was used to label the nucleus. Obtained images were generated by Zeiss LSM 700 confocal microscope. ImageJ was used to quantify the intensity of the immunofluorescence staining in the area defined by DAPI staining as previously described (Jensen, 2013).

### Western Blot

Nuclear and cytoplasmic protein fractions were isolated from confluent cultures using NE-PER kit (Nuclear and Cytoplasmic Extraction Reagents, #78833, Thermo Fisher Scientific) according to the manufacturer’s instructions. Obtained extracts were separated by SDS-PAGE and transferred to nitrocellulose membranes (Trans-BlotTurboTransfer pack, Bio-Rad). After blocking in 5% milk in 1% Tween-20-tris-buffered saline, the membranes were incubated with the primary antibodies at 4°C overnight. Membranes were washed and incubated with horseradish peroxidase-conjugated secondary antibodies (1:1000) for 2 h at room temperature. Blots were developed using enhanced chemiluminescence substrate (Thermo Fischer Scientific).

### Length and Volumetric Analysis of Limb Bones

Limb bone µCT data was acquired using a µCT (SkyScan 1272, Bruker) with the following data acquisition parameters: 60–70 kV, 142–166 μA, isotropic voxel size 4.5–5.0 µm. Three-dimensional (3D) reconstructions and length and volume measurements were performed with the software Avizo 9.0 (Thermo Fisher Scientific), using the 3D length and material statistics tools (the latter after segmentation with the magic wand tool).

### Statistical Analysis

One-way ANOVA tests were used for the analyses of limb bones. Student’s *t*-test was used for skin analyses. Two-way ANOVA tests were used for the analysis of the intensity of Gli protein immunostainings.

## Results

### Loss of Ptch2 Affects Organ Size and Regeneration

**FIGURE 1:**
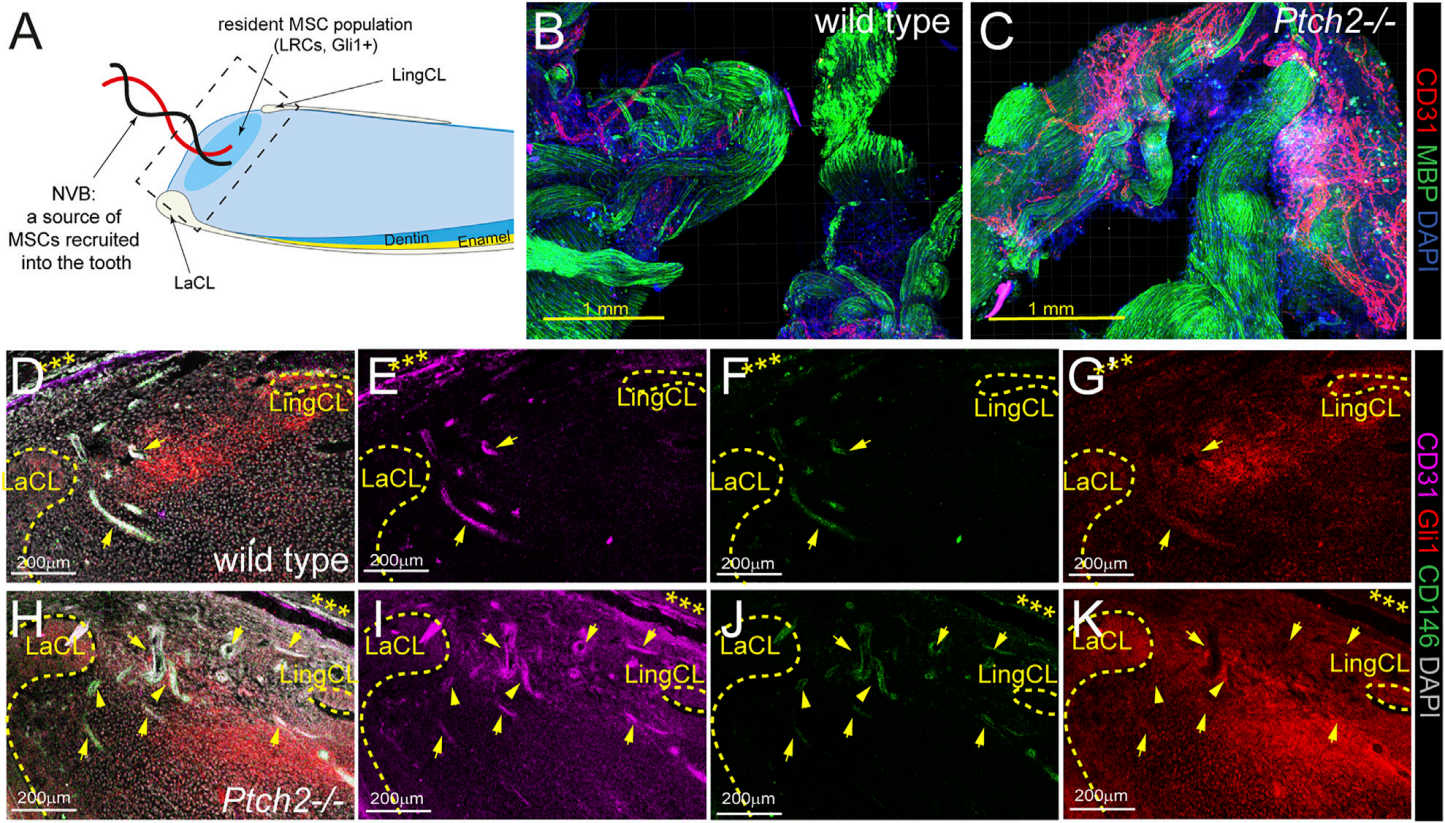
Analysis of SC populations in *Ptch2* ^*−/−*^ incisors. **(A)** Schematic illustration of sagittal section of mouse incisor (LaCL = labial cervical loop, LingCL = lingual cervical loop). Square with dashed line shows the area of sections in D-K. **(B,C)** Immunolabeling for CD31 (red) and MBP (green) in the wholemount wild-type and *Ptch2* ^*−/−*^ neurovascular bundles. **(D–K)** Immunofluorescence labeling of CD31 (pink), GLI1 (red), and CD146 (green) in frozen sections of the incisor proximal ends isolated from 4-week-old wild-type and *Ptch2* ^*−/−*^ animals. Cervical loops are indicated by yellow dashed line. Arrows indicate blood vessels identified by CD31 staining. Asterisks indicate peripheral nerve. Scale bars: yellow = 1 mm, white = 200 μm.

In the adult mouse incisor Ptch2 is expressed exclusively in the epithelium. Loss of *Ptch2* increases the differentiation rate of epithelial SCs into ameloblasts and increases deposition of mineralized matrices (Binder et al., 2019). Incisor growth, however, also depends on MSC populations, both resident and those recruited via neurovascular bundle (NVB) (Figure 1A) (Feng et al., 2011; Kaukua et al., 2014; Zhao et al., 2014). 3-4-week-old *Ptch2* ^*−/−*^ NVBs were grossly comparable to the NVBs of the wild-type littermates, as shown by the expression of Myelin Basic Protein (MBP) (Figures 1B,C). However, *Ptch2* ^*−/−*^ NVBs at this stage show increased vascularization identified by the expression of endothelial marker CD31 (Figures 1B,C). Similarly, at this stage, *Ptch2* ^−/−^ incisors showed increased number of blood vessels in the dental pulps, compared to their wild-type littermates (Figures 1D–K). The average number of blood vessels found in *Ptch2* ^−/−^ dental pulp (*n* = 3) was 13.4 ± 2.07/section in comparison to 9 ± 1/section observed in the wild-type (*n* = 3). Increased vascularization implies higher numbers of recruited, blood-vessel-associated MSCs, like perivascular cells or pericytes identified by the expression of CD146, Gli1, and αSMA, among others (Feng et al., 2011; Zhao et al., 2014; Vidovic et al., 2017). We confirmed increased numbers of CD146^+^ and Gli1^+^ cells by immunostaining of the proximal end of the incisor (compare Figures 1F,G to Figures 1J,K, respectively).

**FIGURE 2:**
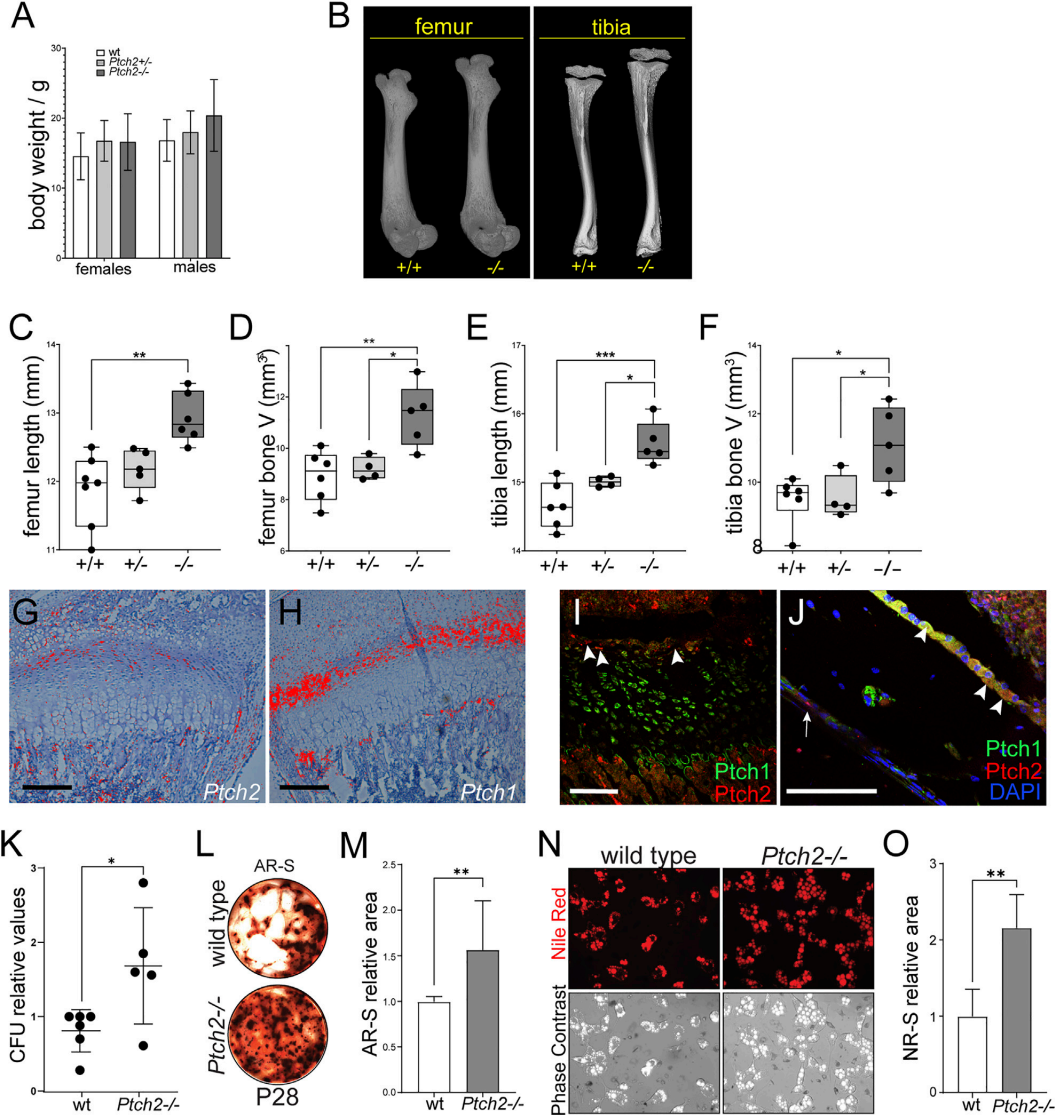
Loss of *Ptch2* affects bone size and SC potential. **(A)** Body weight of P28-35 wild-type, *Ptch2* ^*+/−*^ *,* and *Ptch2* ^*−/−*^ mice**. (B–F)** QuantificationοfμCT analysis of P28-33 femora and tibiae isolated from wild-type, *Ptch2* ^*+/−*^, and *Ptch2* ^*−/−*^ littermates. **(G,H)** Radioactive *in situ* hybridization in 2-week-old tibiae detecting *Ptch2* **(G)** and *Ptch1* **(F)**. **(I,J)** Immunostaining for Ptch1 (green) and Ptch2 (red) in the growth plate **(I)** and cortical bone **(J)** of 4-week-old tibiae. Arrowheads indicate Ptch2^+^ cells in the resting zone of the growth plate **(I)** and endosteum **(J)**. Arrow in **(J)** indicates Ptch2^+^ cells in the periosteum layer. **(K–O)** Analysis of the mineralization **(K–M)** and adipogenic **(N,O)** differentiation potential of bone marrow stromal stem cells. Alizarin Red Staining **(L)** of terminated cultures at day 21 of culture and Nile Red imaging **(N)** of live adipocytes in 14-day-old cultures of bone marrow stromal stem cells isolated from 8-week-old animals. P-values: *p ≤ 0.05, **p ≤ 0.01, ***p ≤ 0.001. Scale bars 100 μm.

By 4 weeks of age, *Ptch2* ^*−/−*^ mice exhibited a trend of increased body weight, albeit not statistically significant (Figure 2A). μCT analyses revealed size-related changes of skeletal tissue, including longer *Ptch2* ^*−/−*^ femora (Figures 2B,C) and tibiae (Figures 2B,E) and increased total bone volume (Figures 2D,F). In the long bones both Hh receptors are partially co-expressed at the mRNA and protein levels (Figures 2G–J). In comparison to broadly expressed Ptch1 (Figure 2H), Ptch2 is expressed in a narrow domain, in the resting zone of the growth plate (Figures 2I,J), periosteum layer (arrow in Figure 2J), and cells lining the endosteal side of the bone (arrowheads in Figure 2J). These locations are considered putative niches for the MSCs in the bone (Ambrosi et al., 2019), which suggests that the observed phenotype could relate to the effect of Ptch2 on MSCs. Correspondingly, bone marrow stromal cells isolated from the 4-week-old *Ptch2* ^*−/−*^ long bones exhibited increased colony forming potential (CFU), mineralization, and adipogenic potential (Figures 2K–O). These analyses suggest that Ptch2 regulates skeletal MSC populations in the long bones, and, as shown in the incisor tooth, acts as an inhibitor of their differentiation activity.

**FIGURE 3:**
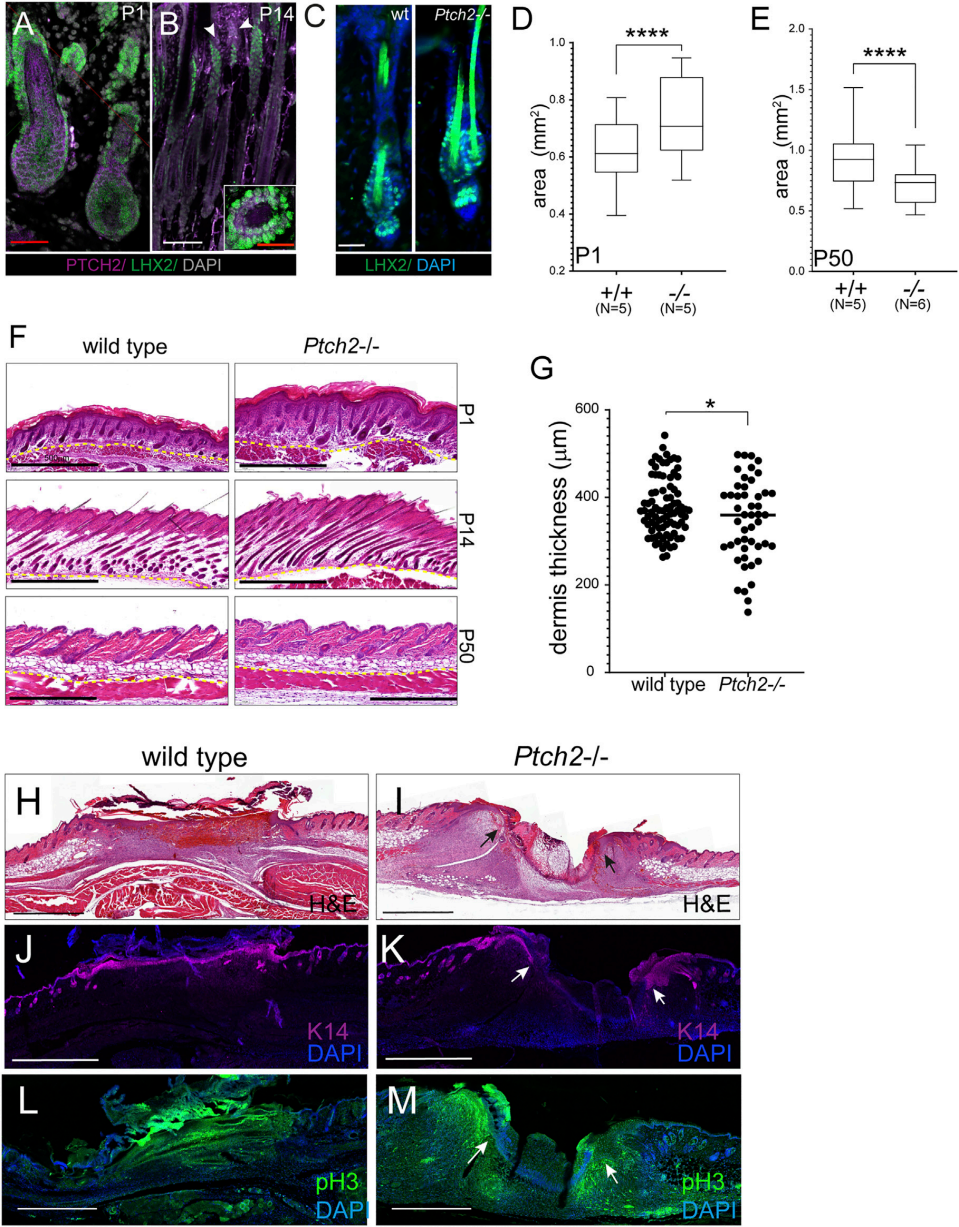
Effects of Ptch2 abrogation on skin thickness and wound healing. **(A, B)** Immunofluorescence staining for Ptch2 (purple) and Lhx2 (green) in wild-type sections of P1 **(A)**, P14 **(B)**. White arrowheads indicate Ptch2 expression. Inset in **(B)** is a cross-section through P50 hair follicle processed with Lxh2 and Ptch2 immunostaining. **(C)** Immunofluorescence staining for Lhx2 (green) in adult (P50) wild-type and *Ptch2* ^*−/−*^ skin. **(D, E)** Quantitative analysis of skin thickness in wild-type and *Ptch2* ^*−/−*^ at P1 and P50. **(F)** H&E staining of paraffin sectioned skins obtained from wild-type and *Ptch2* ^*−/−*^ mice at indicated time points. **(G)** Quantitative analysis of dermis thickness in wild-type and *Ptch2* ^*-/*^. Scale bar red = 50 μm, white = 100 μm, black = 0.5 mm, ****p < 0.0001. **(H–M)** H&E staining **(H,I)** and immunofluorescence against Keratin 14 **(J,K)** and pHH3 **(L, M)** of sectioned wild-type and *Ptch2* ^*−/−*^ skin wounds at day 7 post-wounding. Scale bars (black and white) = 0.5 mm. Arrows indicate wound edges in the *Ptch2* ^*−/−*^ wounds.

Another organ affected by the absence of *Ptch2* is skin. Previous studies have shown that most of the *Ptch2* hypomorph mutants display a normal skin phenotype, although some males can develop alopecia, ulcerations, or epidermal hyperplasia later in life (Nieuwenhuis et al., 2006). During early skin morphogenesis, Ptch2 is broadly expressed in the skin (Figure 3A), but at later stages, Ptch2 expression is limited to fewer cells both in the epithelium and the mesenchyme (Figure 3B) (Motoyama et al., 1998; Adolphe et al., 2014; Joost et al., 2020). The scattered Ptch2^+^ cells found within the mesenchyme (dermis) during late morphogenesis are likely fibroblast and fibroblast-like cells (Joost et al., 2020), while in the epithelium Ptch2 expression is restricted to the hair follicle bulge, adjacent to the secondary hair germ, where it partially co-localizes with hair bulge SC marker Lhx2 (Figure 3B) (Rhee et al., 2006; Mardaryev et al., 2011). Loss of Ptch2, did not affect the epithelial compartment of the skin, as we observed no change in the morphology of the hair follicles, or the expression of hair follicle SC markers, like Lhx2, in the adult *Ptch2* ^*−/−*^ skin (Figure 3C). However, we observed thicker skin in *Ptch2* ^*−/−*^ mice throughout early postnatal morphogenesis (Figures 3D,F) and until the first resting phase (P50), at which *Ptch2* ^*−/−*^ skin was thinner (Figures 3E,F). Interestingly, decreased thickness of adult *Ptch2* ^*−/−*^ skin is entirely due to decreased thickness of the dermis (Figure 3G).

We performed a skin wounding experiment to test if abrogation of *Ptch2* affects skin reparative potential (Figures 3H–M). Within 7 days, wounds on 8-week-old wild-type animals (N = 3) completely epithelialized (Figures 3H,M) (Biggs et al., 2015; Aragona et al., 2017), This contrasted with *Ptch2* ^*−/−*^ wounds (N = 3), which showed distinct epithelial fronts (arrows in Figures 3I,K) separated by inflammatory cells and a thin strip of mesenchymal cells. Analysis of cell proliferation showed that the majority of the proliferating cells localized to the leading edge of wound closure in both epithelium and the mesenchyme of *Ptch2* ^*−/−*^ skins (arrows in Figure 3M). To determine if delayed wound closure was due to altered migration of highly proliferative *Ptch2* ^*−/−*^ skin mesenchyme, we performed *in vitro* scratch assay on fibroblasts isolated from adult *Ptch2* ^*−/−*^ and wild-type skins. The motility of the mutant fibroblasts was comparable to wild-type (15.12 ± 0.4 μm/h vs. 15.23 ± 2.2 μm/h, respectively), and mutant scratch wounds were closing in a similar temporal manner (not shown).

### Ptch2 Transduces Hh Signaling Through Regulation of Gli1

**FIGURE 4:**
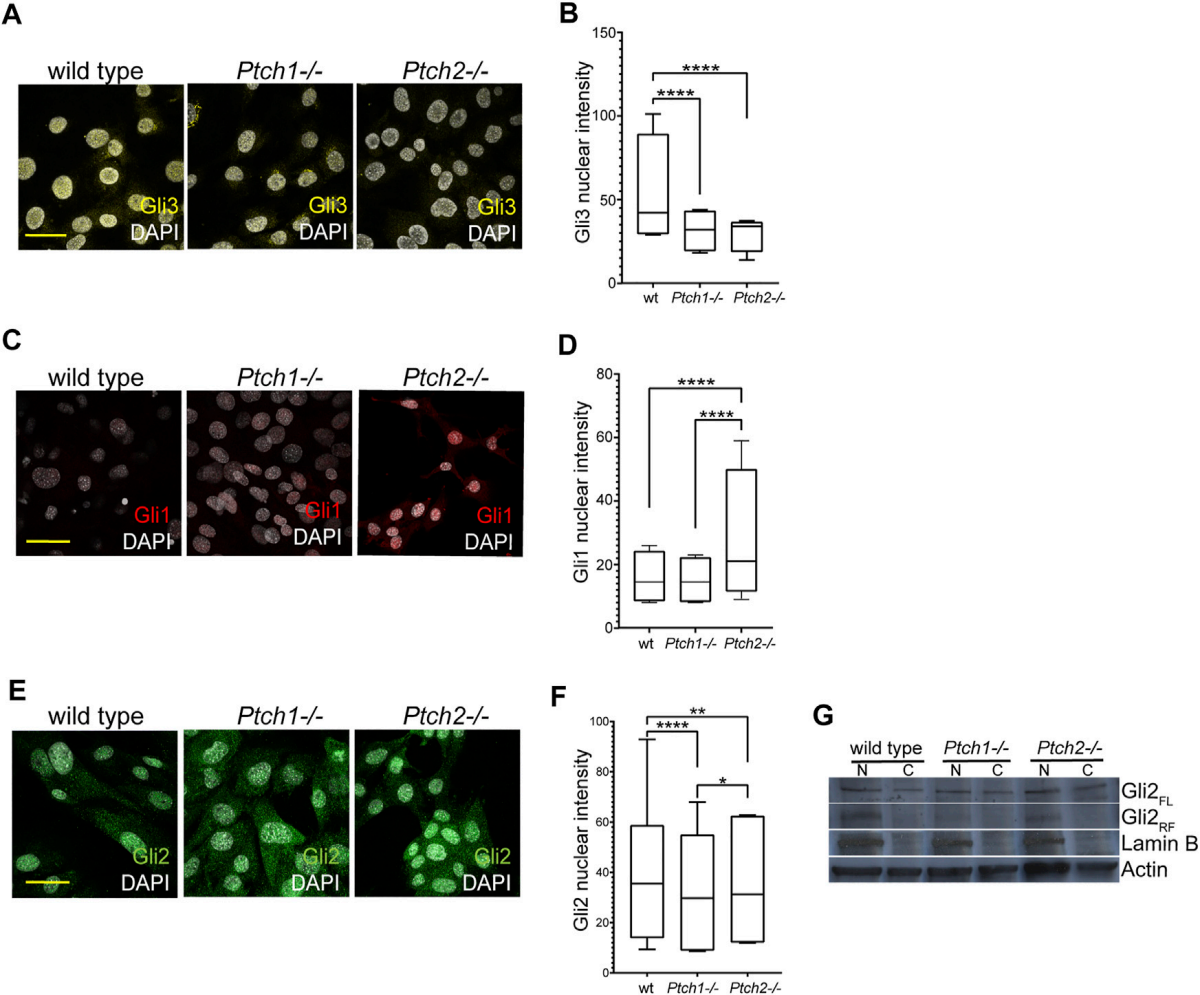
Analysis of Ptch receptor-dependent inhibition of Hh signaling. **(A,C,E)** Immunocytochemistry detection of Gli3 **(A)**, Gli1 **(C)** and Gli2 **(E)** in the wild-type, *Ptch1* ^*−/−*^ *,* and *Ptch2* ^*−/−*^ MEFs. Scale bar 50 μm. **(B,D,F)** Quantification of the intensity of the nuclear GLI3 **(B)**, GLI1 **(D)**, and GLI2 **(F)** in wild-type, *Ptch1* ^*−/−*^, and *Ptch2* ^*−/−*^ MEFs. **p ≤ 0.005, ***p ≤ 0.0005, ****p < 0.0001. **(G)** Western blot showing nuclear (N) and cellular **(C)** expression of Gli2 (FL = full length and RF = repressor form) in wild-type, *Ptch1* ^*−/−*^ and *Ptch2* ^*−/−*^ MEFs.

Observed changes in the *Ptch2* ^*−/−*^ mice indicate an independent role for Ptch2 in Hh signaling, which is supported by data published by us and others, that show independent Ptch2 regulation of genes involved in the cellular hierarchy of the incisor epithelial stem cell niche (Binder et al., 2019), and presence of a non-canonical Hh signaling cascade mediated by Ptch2 (Kim et al., 2020). To further unravel the mechanism through which Ptch2 exerts the independent role, we analyzed the expression and distribution of Gli proteins in Hh responsive cells, namely mouse embryonic fibroblasts (MEFs) that are frequently used for functional *in vitro* studies of Hh pathway (Figure 4). In mice, three Gli proteins (Gli1, 2, and 3) establish a canonical Hh signaling cascade (Hui and Angers, 2011). These proteins share an overlapping activator function, while only Gli3, and to lesser extent Gli2, contribute to repressor function (Hui and Angers, 2011). We analyzed the expression of Gli3, the principal repressor of Hh pathway, in untreated, proliferating cultures of wild-type, *Ptch1* ^*−/−*^, and *Ptch2* ^*−/−*^ MEFs. Compared to wild-type MEFs, Gli3 was significantly reduced in both mutants (Figures 4A,B), which suggests activated Hh signaling in both mutant MEFs. Comparable levels of Gli3 nuclear intensity in the mutant MEFs (Figure 4B) indicate that Ptch receptors inhibit Hh signaling at similar levels. Examination of the expression levels and localization of the Gli1 protein showed significantly increased levels of nuclear Gli1 only in *Ptch2* ^*−/−*^ MEFs (Figures 4C,D), which is opposite to the published *Gli1* mRNA expression in *Ptch1* ^*−/−*^ and *Ptch2* ^*−/−*^ MEFs (Binder et al., 2019). Unexpectedly, Gli2 expression exhibited stronger nuclear staining in wild-type MEFs compared to either mutant cell populations (Figures 4E,F). However, previous studies have shown that Hh pathway activity depends on the ratio of full-length Gli2 protein (the activator form) and repressor form that is generated through proteolytic processing of full-length protein (Pan et al., 2006; Li et al., 2011). Our immunoblotting analysis shows evidence of Gli2 proteolysis in wild-type and *Ptch2* ^*−/−*^ but not *Ptch1* ^*−/−*^ MEFs (Figure 4G), which suggests higher Hh pathway activity in *Ptch1* ^*−/−*^ MEFs compared to the two other MEF cultures. The observed differences in the regulation of Gli1 and Gli2 expression suggest that Ptch2 role in the Hh signaling is independent and mainly exerted through regulation of Gli1.

## Discussion

This brief research report provides evidence that suggests Ptch2 acts as a regulator of growth and regenerative capacity in the postnatal tooth, bone, and skin. Loss of *Ptch2* causes subtle and mild changes in a healthy individual, but this loss can greatly impact an injured organ and may potentially determine the level and capacity for healing and repair. The mechanism behind Ptch2 regulation of the SC populations remains unknown, but here we provide evidence that suggests that Ptch2 governs the regenerative capacity of SC populations through regulation of Gli1.

Our data show that loss of Ptch2 impacts the putative MSC niches in the tooth, which likely contributes to the previously published accelerated incisor growth after injury (Binder et al., 2019). Continuous growth of the mouse incisor is supported by resident MSCs, identified as Gli1^+^ label retaining cells (Zhao et al., 2014), and recruited MSCs which include perivascular pericytes marked by Gli1, CD146, αSMA, and NG2 expression (Feng et al., 2011; Zhao et al., 2014; Vidovic et al., 2017) as well as the glia-derived MSCs (Kaukua et al., 2014). *Ptch2* is expressed in the peripheral nerves (Bajestan et al., 2006; Sharghi-Namini et al., 2006), but it is absent from the tooth mesenchyme (Binder et al., 2019; Krivanek et al., 2020), which indicates that loss of Ptch2 targets mainly recruited MSCs. Therefore, the altered MSC content in the incisor pulp, as well as the increased mineralization in *Ptch2* ^*−/−*^ incisors (Binder et al., 2019) are the result of augmented flow of MSCs into the pulp. The reported low expression of *Ptch1* in the peripheral nerves (Parmantier et al., 1999; Sharghi-Namini et al., 2006) suggests that the Hh signaling regulation of the recruited tooth MSCs is mediated exclusively by Ptch2. Furthermore, increased vascularization in the *Ptch2* ^*−/−*^ NVBs is in line with known proangiogenic and provasculogenic roles of active Hh signaling (Byrd and Grabel, 2004; Lavine et al., 2008).

Concomitantly, in the incisor pulp we observed increases in the number of perivascular MSCs marked by the expression of CD146 and Gli1. Gli1^+^ cells identify MSC population in various tissues, including teeth, craniofacial sutures, and long bones (Zhao et al., 2014; Zhao et al., 2015; Schneider et al., 2017), yet, similar to *Ptch2* ^*−/−*^ mice, Gli1 knockouts show lack of gross phenotype abnormalities (Hui and Angers, 2011). Interestingly, our *in vitro* studies show that Ptch2 transduces Hh signaling mainly through regulation of Gli1 protein activity, which suggests that Ptch2-Gli1 signaling axis regulates the MSC population, as well as the regenerative capacity in the postnatal organs. In this brief report we did not analyze whether Ptch2 and Gli1 colocalize in the putative perivascular MSCs in the NVBs. Previous reports indicate that Gli1 is expressed in the MSCs on the endosteal side of the cortical bone (Schneider et al., 2017), a putative MSC niche, where we also detected Ptch2 expression. This further suggests that Ptch2 regulates MSC population in the bones through Gli1 regulation. Loss of Ptch2 results in increased CFU and differentiation potentials of the MSCs from the bone marrow, which indicates inhibitory role for Ptch2 not only in MSC regulation but also in the regulation of the bone healing and regeneration. Further studies are necessary to determine whether Ptch2 negatively regulates the number, and/or the extent and timing of MSC differentiation in the bone, similar to the injured *Ptch2* ^*−/−*^ incisor (Binder et al., 2019). Previous high resolution transcriptome analyses of the adult skin showed that Ptch2 is expressed in a variety of mesenchymal cells, including dermal papilla, fibroblasts, and fibroblast-like cells, where it is often co-expressed with Gli1 (Joost et al., 2020). However, whether Gli1^+^ fibroblasts in the skin represent the MSCs is not known. Further studies are necessary to test these interesting hypotheses and to also determine how loss of Gli1, alone or in combination with Ptch2, affects the regenerative capacity in the (injured) postnatal organs.

The most exciting changes were observed in the skin, which demonstrated a dynamic change in the thickness and a delay in wound closure. The changes in the thickness likely relate to the spatial-temporal pattern of Ptch2 expression in the skin, where broad Ptch2 expression domain during early skin morphogenesis becomes restricted in the adult skin (Motoyama et al., 1998; Adolphe et al., 2014; Joost et al., 2020). Hair follicle SCs regulate cyclic hair follicle regeneration (Alonso and Fuchs, 2006) and consequently, skin thickness, which dynamically changes throughout different phases of the hair cycle (Hansen et al., 1984). In the adult skin epithelium, Ptch2 expression is restricted to the hair follicle bulge, where it partially overlaps with Lhx2 that regulates early progenitors necessary for hair follicle morphogenesis downstream of Shh (Rhee et al., 2006). The unchanged Lhx2 expression in the adult *Ptch2* ^*−/−*^ skin suggests that changes in the skin thickness are not related to the quantitative changes in the hair follicle SCs, but there is still a possibility that loss of Ptch2 alters the timing of the hair follicle cycle.

Delayed wound closure in *Ptch2* ^*−/−*^ skin is an intriguing finding, and our data indicate that it is not related to loss of proliferative activity or the immobility of *Ptch2* ^*−/−*^ skin cells, but that the probable cause most likely resides within the mesenchymal compartment. The *Ptch2* ^*−/−*^ wounds demonstrated a front of a highly proliferating cells at the wound edges that were incapable of bridging over the wound, and the migration speed of *Ptch2* ^*−/−*^ fibroblasts was comparable to the wild-type control. It is plausible that the differentiation and reparative capacity of MSCs in the *Ptch2* ^*−/−*^ skin is impaired, which is different from the observations in bones and teeth and which implies tissue specific response to the lack of Ptch2 that can potentially be achieved through differential expression of Gli1-3 proteins in these tissues.

Therefore, it would be beneficial to investigate the patterns of Gli1-3 protein expression and their change upon loss of Ptch2 in healthy and wounded tissue. Another plausible explanation for the delayed skin wound closure is the change in the biomechanical properties and the extracellular matrix composition of the *Ptch2* ^*−/−*^ skin that hampers wound closure. A recent study revealed that Shh overexpression in epidermis or constitutive activation of Smo in the dermis results in extensive hair follicle neogenesis in wounds that would otherwise result in scarring (Lim et al., 2018). It would be interesting to see how *Ptch2* ^*−/−*^ wounds proceed beyond the 7-day period analyzed in this study and whether loss of *Ptch2* has any effect on hair follicle formation *de novo*.

Collectively, our data suggest that Ptch2 regulates MSCs in different postnatal organs, and that the subtle, yet intriguing changes observed in these organs result in more striking and impactful changes after wounding. This suggests that Ptch2 regulates postnatal reparative potential. Intriguingly, all the changes observed in *Ptch2* ^*−/−*^ animals occurred in tissues in which a narrow and restricted Ptch2 expression domain overlaps with broadly expressed Ptch1 receptor. A recent study revealed that deletion of *Ptch1* from the stem and progenitor cell populations in the long bone negatively affects bone volume and animal growth (Deng et al., 2019), which contrasts with the effect of *Ptch2* deficiency on the long bones in our study. This suggests that the two receptors independently function within the Hh signaling pathway. Further studies are needed to resolve this hypothesis.

## Data Availability

The original contributions presented in the study are included in the article, further inquiries can be directed to the corresponding author.
